# High-Resolution Ultrasound-Switchable Fluorescence Imaging in Centimeter-Deep Tissue Phantoms with High Signal-To-Noise Ratio and High Sensitivity via Novel Contrast Agents

**DOI:** 10.1371/journal.pone.0165963

**Published:** 2016-11-09

**Authors:** Bingbing Cheng, Venugopal Bandi, Ming-Yuan Wei, Yanbo Pei, Francis D’Souza, Kytai T. Nguyen, Yi Hong, Baohong Yuan

**Affiliations:** 1 Ultrasound and Optical Imaging Laboratory, Department of Bioengineering, University of Texas at Arlington, Arlington, Texas, 76010, United States of America; 2 Joint Biomedical Engineering Program, University of Texas at Arlington and University of Texas Southwestern Medical Center at Dallas, Dallas, Texas, 75235, United States of America; 3 Department of Chemistry, University of North Texas, 1155, Union Circle, #305070, Denton, Texas, 76203, United States of America; 4 Department of Bioengineering, University of Texas at Arlington, Arlington, Texas, 76010, United States of America; University of Glasgow, UNITED KINGDOM

## Abstract

For many years, investigators have sought after high-resolution fluorescence imaging in centimeter-deep tissue because many interesting *in vivo* phenomena—such as the presence of immune system cells, tumor angiogenesis, and metastasis—may be located deep in tissue. Previously, we developed a new imaging technique to achieve high spatial resolution in sub-centimeter deep tissue phantoms named continuous-wave ultrasound-switchable fluorescence (CW-USF). The principle is to use a focused ultrasound wave to externally and locally switch on and off the fluorophore emission from a small volume (close to ultrasound focal volume). By making improvements in three aspects of this technique: excellent near-infrared USF contrast agents, a sensitive frequency-domain USF imaging system, and an effective signal processing algorithm, for the first time this study has achieved high spatial resolution (~ 900 μm) in 3-centimeter-deep tissue phantoms with high signal-to-noise ratio (SNR) and high sensitivity (3.4 picomoles of fluorophore in a volume of 68 nanoliters can be detected). We have achieved these results in both tissue-mimic phantoms and porcine muscle tissues. We have also demonstrated multi-color USF to image and distinguish two fluorophores with different wavelengths, which might be very useful for simultaneously imaging of multiple targets and observing their interactions in the future. This work has opened the door for future studies of high-resolution centimeter-deep tissue fluorescence imaging.

## Introduction

Fluorescence microscopy has broken many physical limits to allow imaging of biological samples and live tissues with unprecedented resolution, depth, contrast, sensitivity, and specificity [1]. Despite significant progresses, fluorescence microscopy is limited to thin samples or superficial tissues (~1 mm) because focusing light into centimeter-deep tissues is extremely difficult [1–2]. However, high-resolution deep-tissue fluorescence imaging is highly desirable for at least the following reasons [3–17]: (1) Much interesting *in vivo* micro-information may deeply locate in tissue, including microcirculation, micro-angiogenesis, micro-lymphangiogenesis, tumor micro-metastasis, and vascularization of implanted tissue scaffolds, etc; (2) Far-red or near-infrared (NIR) light can penetrate centimeter-deep tissue via scattering, although the micro-information is completely blurred; (3) Compared with other imaging modalities such as ultrasound (US), computed tomography (CT), magnetic resonance imaging (MRI), or positron emission tomography (PET), fluorescence imaging stands out in the following respects: high sensitivity, low cost, usage of non-ionizing radiation, feasibility of multi-wavelength imaging, and feasibility of tissue structural, functional, and molecular imaging.

To conduct high-resolution imaging in centimeter-deep tissue via fluorescence, we must address the following fundamental challenges [8–17]: (1) confining the fluorescence emission into a small volume to achieve high spatial resolution; (2) increasing fluorescence emission efficiency and sensitively detecting those photons to compensate for the signal attenuation caused by the small voxel size in high-resolution imaging and by tissue scattering/absorption during the propagation toward the photodetector (i.e., increase signal strength and detection sensitivity to signal photons); and (3) exclusively differentiating signal photons from other background photons to increase signal-to-noise ratio (SNR) and sensitivity (i.e., increase the detection specificity to signal photons and reduce noise).

To address the first challenge, optical focusing has been replaced by with ultrasonic focusing because tissue acoustic scattering is ~1000 times lower than optical scattering [8–16],[18],[19]. Using this method, high-resolution (from microns to hundreds of microns) fluorescence images have been demonstrated at millimeters depth in tissue phantoms [8–10],[12–14]. For example, previously our group developed a technique named continuous-wave ultrasound-switchable fluorescence (CW-USF) [13]. This technique has been demonstrated to achieve high spatial resolution of 290 μm in 8 mm-thick tissue phantoms by using indocyanine green (ICG)-encapsulated poly(N-isopropylacrylamide) (PNIPAM) nanoparticles (NPs) and a continuous-wave USF imaging system. Despite these successes, these technologies face a fundamental barrier: the quick degradation of SNR and sensitivity to an unacceptable level with the increase of the imaging depth, the spatial resolution, or both. This is due mainly to the fluorescence signal’s quick attenuation below the noise level. Accordingly, high-resolution centimeter-deep tissue fluorescence imaging with high SNR and high sensitivity still remains unachievable.

To break this barrier, we must address the challenges described above. In this study, we developed an NIR frequency-domain ultrasound-switchable fluorescence (FD-USF) imaging technique to break these limitations. First, we discovered and synthesized unique USF contrast agents whose fluorescence emission can be switched on or off by a focused ultrasound wave [13],[14]. The emission intensity on-to-off ratio (I_ON_/I_OFF_) can reach >200, which is >100 times higher than that of the current agent with a similar structure (~1.8) [15],[16]. The large value of I_ON_/I_OFF_ is one of the keys to achieve high SNR and high sensitivity in centimeter-deep tissue. Second, we developed a FD-USF imaging system to sensitively detect USF signal while significantly suppressing noise. Last, we adopted a correlation algorithm to differentiate USF signal from noise effectively. With these unique technologies, we demonstrated, for the first time, the feasibility of high-resolution fluorescence imaging with high SNR and picomole sensitivity in centimeter-deep tissue-mimic phantoms and porcine muscle tissues. Compared with previous CW-USF technique, FD-USF technique developed in this study is different in these aspects: (1) the USF contrast agents are new and were synthesized by using a different thermosensitive polymer (pluronic) and a new NIR fluorophore (ADP(CA)_2_), which significantly increased the fluorescence emission efficiency and I_ON_/I_OFF_; (2) the system is a frequency-domain system with 1KHz modulation, which increased the sensitivity of photon detection compared with the previous CW mode system; and (3) a unique correlation algorithm was adopted for this FD-USF system, which enabled one to uniquely identify USF photons, while no specific signal identification algorithms were adopted in CW-USF.

## Results

### Unique USF contrast agents with I_ON_/I_OFF_>200 via a new NIR fluorophore

**Fig 1:**
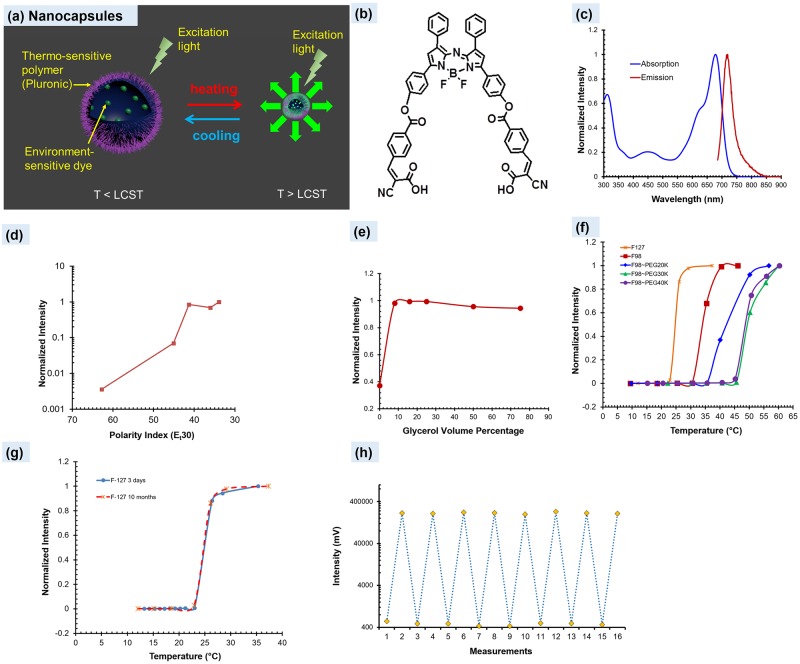
Characterization of ADP(CA)_2_ and ADP(CA)_2_-based USF contrast agents. (a) A scheme displays the principle of USF contrast agents (LCST: lower critical solution temperature). (b) and (c) show the chemical structure, absorption and emission spectra (in dichloromethane) of the environment-sensitive dye, ADP(CA)_2_. (d) and (e) show the fluorescence intensity of ADP(CA)_2_ as function of polarity and viscosity of the solvent, respectively. Five solvents with different polarity index were employed, which are water (62.8), dimethyl sulfoxide (45.1), 1,2-dichloroethane (41.3), 1,4-dioxane (36) and toluene (33.9). A small polarity index represents low polarity. Viscosity was adjusted by varying the volume ratio of glycerol/(ethylene glycol). A high ratio means a high viscosity. (f) The switching relationship between the fluorescence intensity of these ADP(CA)_2_-based USF contrast agents and the temperature: Pluronic-F127 (stars); Pluronic-F98 (squares); Pluronic-F98~PEG20k (diamonds); Pluronic-F98~PEG30k (triangles); Pluronic-F98~PEG40k (solid circles). (g) shows the two switching curves measured at different time from the adopted contrast agent, ADP(CA)_2_-encapsulated Pluronic-F127 nanocapsules. The blue line represents the data acquired three days after the agent was synthesized. The red dashed line represents the data acquired ten months after the agent was synthesized. (h) Repeatable switching of the fluorescence intensity measured from ADP(CA)_2_-encapsulated Pluronic-F127 nanocapsules at low (15°C) and high (35°C) temperatures.

When we encapsulate an environment-sensitive fluorescent dye into a thermo-sensitive nanocapsule, the dye’s fluorescence emission exhibits a switch-like function of the temperature. Fig 1(a) schematically shows the USF concept of the synthesized nanocapsules. When the temperature is below a certain threshold (T_th1_), the nanocapsule exhibits hydrophilicity and provides a water-rich, polar, and non-viscous microenvironment in which the dye shows very low emission efficiency (so-called OFF). When the temperature is above a threshold (T_th2_), the nanocapsule exhibits hydrophobicity and can dramatically shrink and expel water molecules. Thus, the nanocapsule provides a polymer-rich, non-polar, and viscous microenvironment in which the dye shows strong emission (so-called ON). If the transition bandwidth (T_BW_ = T_th2_-T_th1_) is narrow, the fluorescence intensity appears a switch function of the temperature (see Fig 1(f)).

Achieving a high SNR and high sensitivity in centimeter-deep tissue requires a large value of I_ON_/I_OFF_. We synthesized and characterized an extremely environment-sensitive NIR dye whose chemical structure Fig 1(b) displays. The dye is an aza-BODIPY (abbreviated as ADP) derivative. The ADP (core) was chemically reacted with two cyanocinnamic acids (CA) and is therefore denoted as ADP(CA)_2_ (see S1 File for dye synthesis and Figure A in S2 File for the nuclear magnetic resonance (NMR) and mass spectra). ADP(CA)_2_ has a molecular weight of 927.67 g/M and peak excitation/emission wavelengths of 683/717 nm (in dichloromethane, DCM; Fig 1(c)). They are 27-nm red-shifted compared with those of the ADP core (656/690 nm, respectively). Also, the two CAs can provide two carboxyl acid (COOH) groups for conjugation with other units for future use. We found ADP(CA)_2_ to be extremely sensitive to the polarity (Fig 1(d)) and moderately sensitive to the viscosity (Fig 1(e)) of its solvent. Meanwhile, ADP(CA)_2_ is relatively insensitive to pH and KCl ions in the physiological range (pH: 6.8–7.4; KCl:<150 mM) (see Figure B in S2 File). Therefore, we believe it an excellent candidate for USF imaging.

We adopted commercially available thermo-sensitive polymers (Pluronics) and their co-polymers with polyethylene glycol (PEG) to synthesize thermo-sensitive nanocapsules for encapsulating the ADP(CA)_2_: (1) Pluronic-F127, (2) Pluronic-F98, (3) Pluronic-F98~PEG20k, (4) Pluronic-F98~PEG30k, and (5) Pluronic-F98~PEG40k. The diameters of these nanocapsules were found to be in a range of 20–70 nm via transmission electron microscopy (TEM) (see Figure C in S2 File). Fig 1(f) shows how their fluorescence intensity switches according to temperature; S1 Table provides a summary of those contrast agents. Impressively, all the intensity ratios (I_ON_/I_OFF_) are >200, while previously developed agents have I_ON_/I_OFF_ <10 [13],[15],[16],[20]. Fig 1(g) and 1(h) show that the switching property from ADP(CA)_2_-encapsulated Pluronic-F127 nanocapsules has excellent stability and repeatability. We measured the two curves in Fig 1(g) three days and ten months after synthesizing the agent, respectively. The switching curves match very well. To verify whether the nanocapsules can be repeatedly switched on and off, as an example, Fig 1(h) shows the data measured from ADP(CA)_2_-encapsulated Pluronic-F127 nanocapsules at low (15°C) and high (35°C) temperatures. The fluorescence intensity can be repeatedly switched between the two temperatures for at least eight rounds. Based on the data described above, we expect that ADP(CA)_2_-based agents should provide high SNR and sensitivity for deep-tissue USF imaging. Among five different nanocapsules, we used ADP(CA)_2_-encapsulated Pluronic-F127 nanocapsules in the following experiments. The final concentrations of ADP(CA)_2_ and the Pluronic polymers in the injectable agent solution were ~50 μM and ~16.3 mg/mL, respectively. All the above synthesis protocols and measurement methods can be found in Methods and S1 File.

### Sensitive FD-USF imaging system

**Fig 2:**
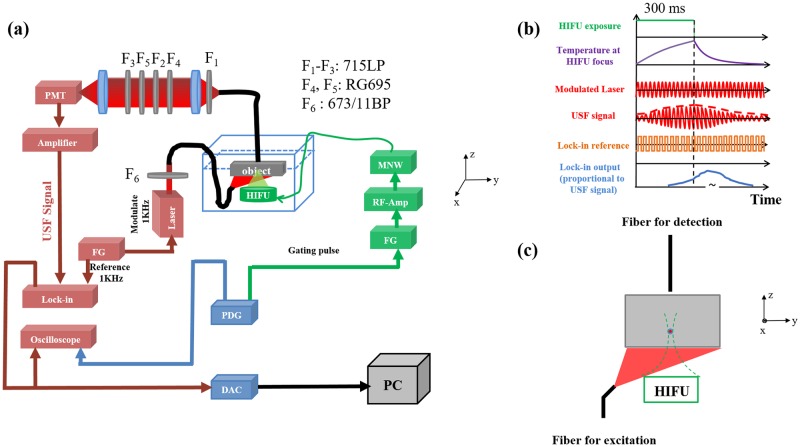
The FD-USF imaging system. (a) The schematic diagram of the USF imaging system. PDG: pulse delay generator; FG: function generator; RF-Amp: radio-frequency power amplifier; MNW: matching network; HIFU: high intensity focused ultrasound; PMT: photomultiplier tube; F1-F5: emission filters; F6: excitation filter; DAC: data acquisition card; PC: personal computer. (b) The schematic diagram of time sequences of six different events in USF imaging, including HIFU transducer gating pulse, temperature change at HIFU focus, modulated laser output, USF signal, lock-in reference and lock-in output. (c) The sample configuration, including the sample, the excitation and emission fiber bundles, and the HIFU transducer.

While excellent USF contrast agents are essential for improving SNR by enhancing fluorescence signal (large I_ON_) and suppressing background fluorescence noise (small I_OFF_), a sensitive imaging system is also critical for improving SNR and sensitivity. Fig 2(a) displays the schematic of the FD-USF system (see the details in Methods). To increase the system’s sensitivity to photons and its specificity to USF signal, we have adopted the following strategies: (1) maximizing the system sensitivity by adopting a lock-in amplifier and a cooled photomultiplier tube (PMT); (2) maximizing the system’s USF signal recognition capability via a correlation algorithm; (3) detecting only the change of the fluorescence signal caused by the ultrasound; (4) suppressing laser leakage by using an emission-light-filtering system (ELFS) in which a collimation tube was combined with multiple emission filters; and (5) minimizing autofluorescence using a far-red excitation laser (671 nm) and NIR dyes.

Briefly, the excitation laser (671 nm) was modulated at 1 kHz. The fluorescence photons were filtered via three interference filters and two absorption filters, then refocused on a cooled PMT. The 1 kHz fluorescence signal was finally detected via a lock-in amplifier (LIA). A high intensity focused ultrasound (HIFU) transducer (2.5 MHz) was focused in the sample to heat its focal volume, switch on fluorescence, and finally induce the amplitude change of the 1 kHz fluorescence. A motorized translation stage was used to scan the sample. A pulse delay generator (PDG) was used to synchronize different sub-systems.

Fig 2(b) and 2(c) show the schematic time sequences of the FD-USF system and the sample setup, respectively. The HIFU exposure time is 300 ms to avoid the thermal diffusion (panel 1), which can be adjusted to induce the temperature to rise around the focal volume (panel 2). In USF imaging, only a few degrees of temperature increase (~2–3 degrees) is applied to minimize the potential of thermal damage. The 1 kHz excitation laser is continuously running (panel 3). The HIFU-induced temperature rise leads to a change in the amplitude of the 1 kHz fluorescence signal (panel 4). After interfering with a phase-locked reference signal (panel 5), the LIA outputs the USF signal (panel 6). The peak of LIA output could be delayed to 1–3 seconds due to the LIA integration function. We subtract the LIA output baseline (I_BG_) from its maximum value (I_Max_) and use the difference (I_USF_ = I_Max_-I_BG_) to represent the USF strength (I_USF_) at that location. I_USF_ can be acquired at each location if the sample or the HIFU focus is scanned to form a USF image. The reason that the USF fluorescence emission does not exactly follow the temperature dynamics is because of two reasons: (1) the existing of the temperature switching threshold of the contrast agents and (2) the electronic signal delay caused by the lock-in amplifier. USF signal occurs only when the temperature is above the threshold. Generally, the tissue background temperature is below the threshold. When ultrasound is applied, the temperature increases and eventually is above the threshold to switch on the agents and give the fluorescence. This will lead to a slight delay of the USF signal compared with the temperature rising. The second factor that causes the delay is related to the response time constant of the lock-in amplifier. In this study, the time constant is variable from 3 to 300 ms. The longer time constant will lead to significant delay and widening of the USF signal appear because of the slow response of the lock-in amplifier.

### Effective signal identification algorithm

**Fig 3:**
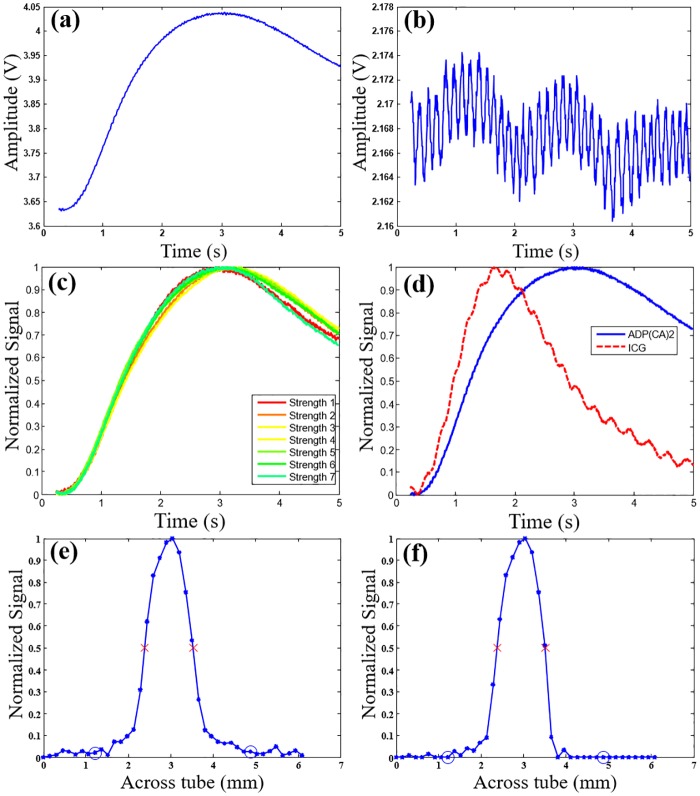
Typical USF signals and the correlation method. (a) and (b) show the typical USF signal and background noise acquired from ADP(CA)_2_-encapsulated Pluronic-F127 nanocapsules in the 8-mm thick tissue USF experiment, respectively. (c) The normalized USF signals (from ADP(CA)_2_-based agents) with different signal strengths (strength 1–7: 171, 239, 290, 310, 325, 306, and 270 mV, respectively) to show that the shape is independent of the signal strength. Different signal strengths were generated by varying the HIFU driving voltage. (d) The normalized USF signals from ADP(CA)_2_-encapsulated Pluronic-F127 nanocapsules (blue solid line) and ICG-encapsulated PNIPAM NPs (red dash line) agents to show that the shape is dependent on the type of the agents. (e) and (f) are the normalized USF profiles of the micro-tube (filled with the ADP(CA)_2_-based agent) before (SNR: 88) and after (SNR: 300) correlation analysis, respectively. The experiment conditions are presented in Methods.

Fig 3(a) and 3(b), respectively, show the typical USF signal and background noise as a function of time. Clearly, the shape of the USF signal does not correlate with the background noise. Currently, we are unclear about the reason of the oscillation in Fig 3(b). Our hypothesis is that it could be some kind of electronic noise or internal noise from the lock-in amplifier. Fig 3(c) shows a total of seven USF signals with different strengths (see figure caption for detailed values) that overlap substantially after normalization, indicating that the shape of the USF signal is also independent of the signal strength. In contrast, the shape of the USF signal depends on the contrast agent type. Fig 3(d) shows the typical USF signals acquired from two types of USF contrast agents: (1) ADP(CA)_2_-encapsulated Pluronic-F127 nanocapsules and, (2) ICG-encapsulated PNIPAM NPs. The two curves have quite different dynamic shapes. Based on our knowledge, we think that the response of USF contrast agents may depend on two factors: 1) the properties of the thermosensitive polymer such as thermal properties and physical structures; and 2) the environment-sensitivity of the encapsulated fluorophores. The data shown in Fig 3(d) were acquired from two different contrast agents as mentioned above. Both the fluorophores and the polymers are different. Therefore, they have different responses. Based on the results described above, we can use a correlation algorithm to differentiate USF signals from background noise and to differentiate different types of USF contrast agents.

Specifically, Eq 1 shows the correlation coefficient (CrC) between a normalized USF signal at any location (*I(t)*) and a normalized USF reference at a selected location (*R(t)*) [21]. The reference was selected from one of the strongest USF signals at a specific location in a USF image (such as the signal in Fig 3(a) or Figure D(a) in S2 File). The total data point of *I(t)* or *R(t)* is *N*. After intense calibration experiments, we defined the algorithm as follows. If CrC<0.3, it is considered noise, and its I_USF_ is set to zero. If 0.3<CrC<0.8, its I_USF_ is multiplied with the cube of CrC to partially suppress its contribution. If CrC>0.8, no modification is applied on its I_USF_. Other details about this algorithm can also be found in S3 File.

$$CrC=\frac{\sum I\left(t\right)R\left(t\right)-\frac{\sum I\left(t\right)\sum R\left(t\right)}{N}}{\sqrt{\left(\sum I{\left(t\right)}^{2}-\frac{{\left(\sum I\left(t\right)\right)}^{2}}{N}\right)\left(\sum R{\left(t\right)}^{2}-\frac{{\left(\sum R\left(t\right)\right)}^{2}}{N}\right)}}$$ (1)

Fig 3(e) and 3(f) display the normalized USF profile of a micro-tube embedded in porcine muscle tissue (thickness: 0.8 cm) that was filled with ADP(CA)_2_-based agent before and after adopting the correlation algorithm, respectively. After the correlation analysis, the signal in targeting area was retained while the signal in background area was suppressed significantly. We applied this correlation method to the following experiments to improve the SNR and achieve multiplex USF imaging.

### Multi-color high-resolution USF images in tissue-mimic silicone phantoms

**Fig 4:**
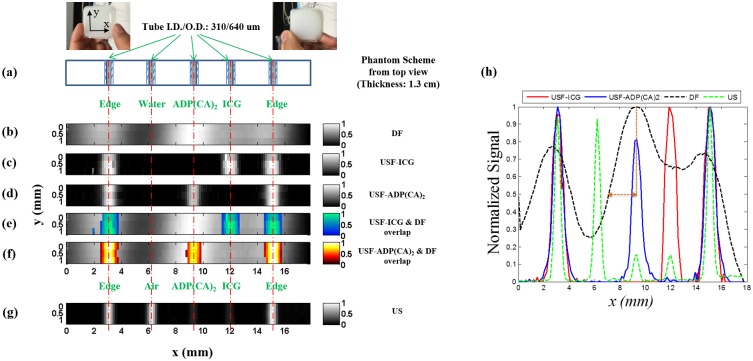
Images of micro-tubes in a tissue-mimic phantom. (a) The photographs and schematic diagram of the tissue-mimic silicone phantom (on x-y plane): front view (left photo), back view (right photo) and the cross section of the micro-tube structure on the x-y plane (the bottom figure in (a)). (b) The image acquired by directly detecting fluorescence (DF) without ultrasound. (c) and (d) show the USF images acquired from ICG-encapsulated PNIPAM NPs (color-1) and ADP(CA)_2_-encapsulated Pluronic-F127 nanocapsules (color-2), respectively. (e) and (f) are the overlapped images between USF images ((c) and (d)) and direct fluorescence image (b). (g) Ultrasound image of the micro-tubes acquired from the same ultrasound transducer (i.e. a C-mode ultrasound image). The three non-edge tubes (the second, third, and fourth tubes) were filled with water (as background control), ICG-based agent (color-1) and ADP(CA)_2_-based agent (color-2), respectively. The two edged micro-tubes (the first and fifth tubes) were used for image co-registration and were filled with the corresponding contrast agent of each modality (see Methods). (h) The normalized profiles along x-axis acquired from different imaging modalities (i.e. the cross section of the five micro-tubes in the silicone phantom). Due to the significant overlap of the tube profiles in the DF image, the two dotted brown lines are used to show how FWHM of the central tube in the DF image (the dashed black line) is estimated. The horizontal dashed brown line with double arrows represents ½ of the FWHM that is ~2.5 mm and therefore the estimated FWHM is ~5 mm.

Five silicone micro-tubes (inner diameter: I.D. = 0.31 mm; outer diameter: O.D. = 0.64 mm) were inserted through the middle plane (x-y; z = 6.5 mm) of a tissue-mimic silicone phantom [22] (μ_a_ = 0.03; μ_s_′ = 3.5 cm^-1^; thickness = 13 mm) (see details in Methods). Fig 4(a) shows two photographs (the front and back) of this phantom and the cross section of micro-tube structure on the x—y plane. These micro-tubes were filled with different contrast agents (see the caption of Fig 4(g)) and imaged using three modalities: directly acquiring fluorescence without ultrasound (DF), USF, and ultrasound (US) (see details in Methods).

Fig 4(b) shows the image acquired from DF. The DF images of the tubes are significantly blurred because of the phantom’s strong light scattering. The estimated full-width-at-half-maximum (FWHM) is ~5 mm for the ADP(CA)_2_ nanocapsules-filled tube (i.e., the third tube). Spatially differentiating the ADP(CA)_2_ nanocapsule-filled tube from the ICG NPs-filled tube (i.e., the third and fourth tube) is difficult. In addition, the spectrum overlap makes both ADP(CA)_2_ nanocapsules- and ICG NPs-filled tubes visible in the image, no matter which emission filters are used (e.g., 715-nm long pass (Fig 4(b)) or 711/25 nm band pass [data not shown]).

Fig 4(c) and 4(d) show the multi-color USF images of ICG NPs (color-1) and ADP(CA)_2_ nanocapsules (color-2), respectively, which can be acquired from a single scan using the same system (Ex:671 nm; Em:715 nm long pass), but are differentiated using the correlation method (the spectrum crosstalk is eliminated). Fig 4(e) and 4(f) show the same USF images overlapped with the DF image. Compared with the DF image, the sizes of the tubes in USF images are significantly reduced (FWHM = 0.88 mm for color-2 tubes and 0.95 mm for the color-1 tubes) and much closer to the true size of the micro-tubes. All the individual micro-tubes can be clearly and correctly resolved.

Fig 4(g) shows the ultrasound image acquired using the same US transducer. The tube size (FWHM = 0.5 mm) represents the spatial convolution of the transducer’s acoustic focus (lateral FWHM = 0.42 mm) with the top inner boundary between the silicon and the liquid (the specific size of the boundary is unknown but should be <I.D. = 0.31 mm). As expected, the ADP(CA)_2_ nanocapsules- and ICG NPs-filled tubes show much lower acoustic contrast than the air-filled tubes and they cannot be differentiated from each other because the acoustic wave is insensitive to fluorophore molecules. For better comparison, Fig 4(h) displays the normalized profiles along the x-axis acquired from different imaging modalities (i.e., the cross section of the five micro-tubes in the silicone phantom).

### USF high-resolution images in centimeter-deep porcine muscle tissues

**Fig 5:**
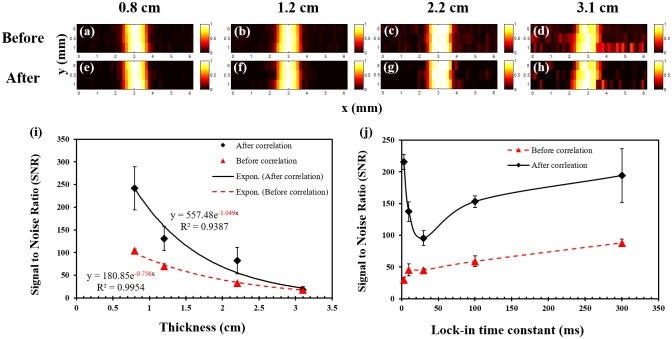
USF images of micro-tubes in porcine muscle tissue samples. (a)-(d) The USF images of the micro-tubes (before correlation) that were embedded into pork muscle tissue samples with thicknesses of 0.8, 1.2, 2.2 and 3.1 cm, respectively. The I.D./O.D. of the tube is 0.31/0.64 mm; (e)-(h) The corresponding USF images processed by the correlation algorithm. (i) The relationship between the SNR and the thickness of the sample before (triangles) and after (diamonds) the correlation processing (error bar: mean±standard deviation). (j) The relationship between the SNR and the LIA time constant before (triangles) and after (diamonds) the correlation processing (error bar: mean±standard deviation).

We used porcine muscle tissue (μ_a_ = 0.06; μ_s_′ = 2.7 cm^-1^) in the USF experiments because of its reasonable acoustic properties and optical properties (see S4 File for details). Fig 5(a)–5(d) show the USF images of micro-tubes that are inserted into porcine muscle tissue with different thickness (0.8, 1.2, 2.2, and 3.1 cm). Fig 5(e)–5(h) show the corresponding images processed with the correlation algorithm. All the micro-tubes can be clearly imaged with a similar FWHM (~1.1 mm). The spatial resolution does not degrade when the thickness increases. Fig 5(i) plots the SNRs as a function of the tissue thickness. Significantly high SNRs have been achieved (17.6–242 after and 18–104 before the algorithm processing). The fitted attenuation coefficient of the SNR versus the tissue thickness (0.756 and 1.049 cm^-1^ before and after the algorithm processing) is slightly smaller or close to the average effective optical attenuation coefficient of porcine muscle tissue (μ_eff_ ≈1 cm^-1^ for 670–900 nm) [23]. This result indicates that (1) the tissue’s optical attenuation is primarily responsible for the SNR reduction as the thickness increases and, (2) increasing the illumination energy of the light and/or ultrasound in thicker tissue can compensate for the tissue’s optical attenuation (see Methods).

Fig 5(j) plots the SNR as a function of the LIA’s time constant (tissue thickness = 12 mm). If we do not apply the correlation algorithm, the SNR increases from 29.9–87.9 when the time constant increases from 3–300 ms. The correlation algorithm dramatically improves the absolute values of SNRs (between 95.4–215.6). The correlation method seems more efficient for short time constants. This is useful to improve USF imaging speed in the future by shortening the time constant without SNR degradation.

For comparison, we also measured SNRs for ICG-based contrast agents. When tissue thickness is <2 cm, SNRs of USF signals acquired from ADP(CA)_2_-based agents are ~3–5 times higher under similar experiment conditions. This improvement is not as high as the improvement in their ON-to-OFF ratios (I_ON_/I_OFF_), primarily because the background noise in USF imaging originates from multiple sources, such as the laser leakage, autofluorescence, and emission from non-100%-OFF fluorophores. Beyond 2 cm, only the ADP(CA)_2_-based agent, because of its high I_ON,_ is capable of imaging the 310μm micro-tube.

## Discussion

The images acquired with US and DF verified the feasibility of USF imaging regarding of target location, size, and shape. Meanwhile, compared with the traditional DF imaging (at centimeters depth), USF improves the spatial resolution >5 times using current setup (2.5 MHz ultrasound). The current system’s resolution is limited mainly by thermal diffusion (see the third paragraph), but in the future, microscopically heating the contrast agents to achieve thermal confinement can further increase it. Once thermal confinement is achieved, adopting an ultrasound transducer with a high frequency and a small f-number can further significantly increase the resolution.

Current USF technology can sensitively and specifically detect USF fluorophores (ADP(CA)_2_) with a concentration of ~50 μM at a depth of several centimeters, using a laser and a HIFU below the safety thresholds (laser intensity: 0.21–3.19 mW/cm^2^; HIFU’s mechanical index: 1.7 when imaging depth <30 mm, and 2.19 when imaging depth >30 mm). In literature there are two commonly used methods to quantify imaging sensitivity. One is fluorescence concentration and another one is fluorophores per voxel. When spatial resolution is high, the latter (fluorophores per voxel) can more accurately reflect the true detection capability (i.e. sensitivity). Because USF imaging is a high resolution imaging modality, the latter one is adopted in our manuscript. Because of the high spatial resolution and the adoption of micro-tubes, the ADP(CA)_2_-occupied volume in a single voxel in this study is as small as ~68 nanoliters (assuming the volume is a cylinder with a diameter of 0.31 mm (I.D.) and a length of 0.9 mm [the FWHM of the USF image]). Thus, the corresponding number of ADP(CA)_2_ molecules in this volume is as low as ~3.4 picomoles. Note that the sensitivity of picomole per voxel (not picomolar, which is concentration) is calculated (not measured) based on the concentration of fluorophores and the volume of tube within a single ultrasound focal volume. This is a reliable and convenient method to estimate the sensitivity because all the needed parameters can be accurately measured, including the concentration and volume. By focusing a small ultrasound beam onto a micro-tube, we do generate a small volume of ~68 nanoliters (as mentioned above). To estimate how many fluorophore molecules in this small volume, we used the maximum fluorophore concentration (~50 μM or ~16.3 mg/mL, which is measured at the beginning of the synthesis of contrast agents. Note that the actual concentration should be lower than this number, but it is difficult to estimate after synthesis). By multiplying the volume and the concentration, we found the fluorophores in this small volume is ~3.4 picomoles. By comparison, conventional fluorescence molecular tomography (DF-based imaging) detects with difficulty <10 picomole molecules of a commercially available dye (AF680) in a mouse phantom at a depth of 11 mm (i.e., a thickness of 22 mm when converting into a transmission geometry) [24]. In FD-USF imaging, further decreasing the concentration of the USF agents is possible but sacrifices intensity and SNR. For example, when the agent’s concentration drops to 50%, the fluorescence intensity decreases 44% and the SNR decreases 32%.

To quantify the point spread function (PSF) and the equivalent spatial resolution of the FD-USF imaging, the profile of the actual tube was de-convolved from the acquired USF profile. The result was considered to be the PSF. The FWHM of the PSF was calculated as ~0.9 mm as the equivalent spatial resolution. Usually the spatial resolution of conventional fluorescence diffuse optical tomography (FDOT) is poor (around 2–5 mm) at a depth of centimeters. However, USF can reach acoustic resolution (about 0.9 mm using a 2.5 MHz ultrasound transducer), which is several times higher. The FWHM of the transducer’s lateral acoustic profile was defined by the lateral focal size of the HIFU transducer (with a central frequency of 2.5 MHz and f-number of 0.83), which was measured ~0.42 mm. The estimated FWHM of the PSF (~0.9 mm) is ~2 times larger, larger also than what we have observed in our previous continuous wave (CW) mode system (0.29 mm) [13]. Current USF resolution is ~2 times worse than the lateral acoustic focal size of the HIFU transducer and ~3 times worse than the resolution our previous study reported, where we had adopted a CW mode system. Thermal diffusion is the main cause of this resolution loss. In the current system, although the ultrasound exposure time remained at 300 ms, USF photon detection required a few seconds because we have adopted the lock-in amplifier. In this study, we set the total data acquisition time at each location to five seconds—much longer than the tissue thermal diffusion time constant (~360 ms) in the current setup. Accordingly, thermal confinement is no longer satisfied and the spatial resolution is degraded, compared to the acoustic focal size and the spatial resolution in the CW mode. However, the gain is the improved sensitivity, SNR, imaging depth, and low concentration of the contrast agent.

Currently, it takes ~20 minutes to acquire one high-resolution USF image. The low speed is caused mainly by the time required for heating and cooling the tissue to minimize the thermal diffusion effect between two adjacent locations. Microscopically heating only USF contrast agents rather than the tissue itself, would dramatically reduce heating and cooling time; we could do this, for example, by attaching current USF nanocapsules to micro-/nano-bubbles where bubbles can absorb acoustic energy to function as a heating source. Correspondingly, using the correlation method could reduce the LIA time constant to milliseconds without sacrificing much SNR (Fig 5(j)). Adopting an ultrasound transducer array with electronic scanning capability to replace the mechanical scanning would help reduce the scanning time. Last, we can develop a smart scanning method to ensure adequate spatial separation of any two sequential heating events and adequate temporal separation of any two heated spatially adjacent locations. Thus, waiting for the tissue to cool down at one location before scanning to the next becomes unnecessary.

To push this technology for *in vivo* applications, the following future directions should be pursued. (1) Optimize current or develop new synthesis strategies for high-threshold (T_th1_>37°C) USF contrast agents; currently, their synthesis yields and shelf lifetime are low. (2) Explore more USF fluorophores to cover the 750–900 nm NIR spectrum for multiplex imaging and even deeper tissue imaging. (3) Significantly increase the imaging speed, minimize artifacts caused by animal motion, and possibly catch dynamic events. (4) Achieve relatively uniform resolution along both lateral and axial directions for three-dimensional imaging. (5) Further improve system sensitivity by increasing photon-collection efficiency and adopting more efficient technologies to suppress the background photons. (6) Modify the system suitably for scanning a live animal, which is currently difficult because of the uneven mouse body, the animal’s motion, and the limited imaging speed. (7) Apply USF for *in vivo* studies. Fortunately, all the above-mentioned challenges are solvable (see S5 File). Therefore, with these improvements, FD-USF will be a powerful fluorescence high-resolution imaging technology with high SNR and high sensitivity for centimeter-deep tissues, useful for many biomedical applications.

## Conclusion

In this study, we achieved high-resolution fluorescence imaging in centimeter-deep tissue phantoms with high SNR and picomole sensitivity by using a series of newly developed technologies. We have synthesized and characterized a NIR extremely environment-sensitive fluorophore, ADP(CA)_2_, and a family of USF contrast agents based on this dye. These USF contrast agents have very high fluorescence intensity on-to-off ratios. We developed a FD-USF imaging system to sensitively detect USF photons and suppress background noise efficiently. Also, we adopted a correlation algorithm to uniquely differentiate USF signals from background noise to further improve the SNR and differentiate one type of USF contrast agent from another. With the help of the correlation algorithm, we also demonstrated multiplex USF fluorescence imaging, which will, in the future, be very useful in simultaneously imaging multiple targets and their interactions that are usually difficult for non-optical technologies.

## Methods

### Characterization of the dye and the USF contrast agents

In S1 File, we have provided the adopted materials and the synthesis protocols of the dye and the USF contrast agents. To measure the dye environmental sensitivity, we prepared a stock solution by dissolving ADP(CA)_2_ in methanol at a concentration of 20 mg/mL (21.5 mM). For each experiment, we dissolved a small portion of this stock solution into different solvents. (1) *Polarity*: we selected the five solvents with different polarity indexes [25]. (2) *Viscosity*: we prepared eight solvents with different viscosities by mixing glycerol and ethylene glycol at different volume ratios: 0/100, 8/92, 16/84, 25/75, 50/50, 75/25, 90/10, and 100/0. The final concentration of ADP(CA)_2_ in each solvent was 8.6 nM. The fluorescence measurement system was similar to the one we used in our previous report (Ex: a 655-nm laser; Em: a 711/25-nm band-pass emission filter) [20].

### FD-USF imaging system

In Fig 2(a), a diode laser (MLL-FN-671, 671 nm, Dragon Lasers, Changchun, Jilin, China) was the excitation light source. The laser was modulated at 1 KHz via a function generator (FG, 33220A, Agilent, Santa Clara, CA, USA). At the same time, the same FG generated a phase-locked signal and sent it to the LIA (SR830, Stanford Research Systems, Sunnyvale, CA, USA) as its reference signal. We used a band pass filter F_6_ (FF01-673/11-25, Semrock, NY, USA; central wavelength: 673 nm; bandwidth: 11 nm) as an excitation filter to clean up any undesirable sideband components of the diode laser, which was located in the pass band of the emission filters. To achieve the best SNR (maximally suppressing laser leakage), we adopted three long pass interference filters (F_1_ to F_3_; FF01-715/LP-25, Semrock, Rochester, NY, USA; edge wavelength: 715 nm) and two long pass absorptive glass filters (F_4_ and F_5_; FSR-RG695, Newport, Irvine, CA, USA, cut-on 695 nm) and positioned them as shown in the figure after intensive experimental trials. We used two NIR achromatic doublet lenses (AC-254-035-B, Thorlabs, Newton, NJ, USA) to collimate the fluorescence photons so that the interference filters could best reject the excitation photons and to focus the filtered photons onto a cooled and low-noise PMT (H7422-20 driven by a high-voltage source C8137-02, Hamamatsu, Japan). The signal was further amplified by a low-noise current preamplifier (SR570, Stanford Research Systems, Sunnyvale, CA, USA) and was then fed into the LIA as the input signal. The output signal of the LIA was acquired by a multichannel oscilloscope (MSO4102B-L, Tektronix, Beaverton, OR, USA) and a data acquisition card (BNC-2110, National Instruments, Austin, TX, USA), after which the data was sent to a PC for offline processing. A gated sinusoidal wave signal with a central frequency of 2.5 MHz was generated by the second FG (33220A, Agilent, Santa Clara, CA, USA) and further amplified by the RF power amplifier (325LA, E&I, Rochester, NY, USA). The amplified signal was input to the MNW to drive the HIFU transducer (H108, Sonic Concepts Inc., Bothell, Washington, USA). The HIFU transducer was focused on the micro-vessel. We used the PDG (P400, Highland Technology Inc., San Francisco, CA, USA) to synchronize the entire system. We mounted the HIFU transducer on a manual three-dimensional translation stage for initial positioning and mounted the sample on a motorized three-dimensional translation stage for subsequent scanning. After initially localizing the HIFU transducer on the vessel, we performed a raster scan.

### Definition of SNR of a USF image or profile

In this study, we defined and calculated the SNR of a USF image (or a profile) based on the following rules. First, we plotted the USF profile along the x direction at each y location on a USF image. We found and defined the profile’s peak strength of the profile as signal strength (S). Second, we defined a range on the x-axis, centered at the peak location of the profile and with a width of three times of the FWHM of the profile, as the region for potentially detecting USF signals. We defined the area outside this region as the background region and defined standard deviation of all the data measured in the background region as noise strength (N). Third, we defined the ratio of S to noise strength (S/N) as the SNR of the profile. Fourth, we defined the average and the standard deviation of the SNRs of all the profiles on each USF image as the SNR of the USF image and the corresponding error.

### Multicolor USF, DF, and US imaging in silicone phantom

We constructed the tissue-mimic phantom using silicone (to mimic tissue’s acoustic properties) doped with TiO_2_ (to mimic tissue’s optical scattering properties) [22]. We used the two edged micro-tubes (the first and fifth tubes) for image co-registration and filled them with the corresponding contrast agent of each modality: air for US imaging, ICG-based agent for color-1 USF imaging, ADP(CA)_2_-based agent for color-2 USF imaging, and ADP(CA)_2_-based agent for DF imaging. Our procedures for DF imaging and ultrasound imaging were similar to those we adopted in our previous paper [13].

### USF imaging in porcine muscle tissues

We purchased the tissues from a local grocery store (99 Ranch Market, Plano, TX, USA) and cut them into different thicknesses. The experimental parameters for the four thicknesses (0.8, 1.2, 2.2, and 3.1 cm) were as follows: laser power: 2.5, 2.5, 2.5, and 38.64 mW; corresponding laser intensity right before entering the tissue: 0.21, 0.21, 0.21, and 3.19 mW/cm^2^; LIA sensitivity: 500, 500, 200, and 200 mV nA; HIFU driving voltage: 80, 80, 80, and 100 mV (the estimated mechanical index is 1.7, 1.7, 1.7 and 2.19). For all experiments, the LIA time constant was set as 300 ms. All the experimental parameters used in Fig 3 were the same as those in the 8-mm thick tissue experiment described here.

## Supporting Information

S1 File Materials and synthesis protocols. (DOCX)

S2 File Supporting figures. (DOCX)

S3 File Effective signal identification algorithm. (DOCX)

S4 File Reason for choosing porcine muscle tissue. (DOCX)

S5 File Further discussion about future directions. (DOCX)

S1 Table Summary of USF contrast agents. λ_ex_ is the wavelength of the excitation laser in the measurements (655 nm), and λ_em_ is the central wavelength of the band pass emission filter. The number of 25 in the parentheses is the bandwidth of the emission filter. The definitions of the other three parameters are the same as those in our previous publication^3^.
(DOCX)
