# Supplementary material for: High-Resolution Ultrasound-Switchable Fluorescence Imaging in Centimeter-Deep Tissue Phantoms with High Signal-To-Noise Ratio and High Sensitivity via Novel Contrast Agents
Source: PLoS One. 2016 Nov 9;11(11):e0165963. doi: 10.1371/journal.pone.0165963 (PMC5102469; doi:10.1371/journal.pone.0165963)
Supplement: S1 File — (DOCX) [file pone.0165963.s001.docx]

**Materials**

Tetrabutylammonium iodide (TBAI), 4-dimethylaminopyridine (DMAP), dicyclohexylcarbodiimide (DCC), 4-carboxy benzaldehyde, 4,4’-azobis(4-cyanovaleric acid) (ACA), 1-ethyl-3-(3-dimethylaminopropyl) carbodiimide (EDCI), piperidine, cyanoacetic acid, acetonitrile and N,N-dimethyl formamide (DMF), and dimethyl sulfoxide (DMSO, anhydrous) were purchased from Sigma-Aldrich Corporate (St. Louis, MO, USA). Pluronic F127 and F98 were obtained from BASF (Florham Park, NJ, USA). Methoxyl PEG carboxylic acid (PEG-COOH) products (molecular weight (MW)=20000, 30000, and 40000) were purchased from Nanocs Inc. (New York, NY, USA) and bulk solvents dichloromethane, hexane, and methanol were purchased from Fisher Chemicals (Pittsburgh, PA, USA). All chemicals were used as received.

**Synthesis protocols**

**(1) Synthesis of ADP(CA)_2_.** Scheme A depicts the synthetic procedure, which consists of two steps. First, we dissolved 4-carboxybezaldehyde (135 mg, 1.27 mmol) in (30 cm^3^) DMF to which we added EDCI (191.44 mg, 1.53 mmol) at 0 ^°^C under N_2_, followed by compound **1** (135 mg, 0.255 mmol), after which the mixture was stirred for twenty-four hours. We synthesized compound **1** according to our earlier published method [1]. We then removed the solvent under reduced pressure, dissolved the residue in CH_2_Cl_2_, and washed the mixture with water. We then separated and dried the organic layer over Na_2_SO_4_ and evaporated the solvent. We purified the residue using column chromatography on silica gel with CH_2_Cl_2_: hexanes (1:1) to produce compound **1a**. The yield was 100 mg (49%); 1H NMR (400 MHz, CDCl3) δ=10.17 (s, 2H), 8.39 (d, 2H), 8.14 (d, 2H), 8.11–8.03 (m, 10H), 7.52–7.41 (m, 8H), 7.38 (d, 2H), 7.11 (s, 1H), 7.01 (s, 1H), 6.96 (d, 2H).

Second, we dissolved compound **1a** (100 mg, 0.126 mmol) in (30 cm^3^) acetonitrile and added cyanoacetic acid (64 mg, 0.752mmol) followed by the addition of piperidine (0.5 ml, 0.005mmol), refluxing the resulting mixture overnight under nitrogen. We then removed the solvent under reduced pressure, purifying the residue by column chromatography on silica gel with CH_2_Cl_2_: methanol (4:1) to give the compound ADP(CA)_2_. The yield was 60 mg (51%); 1H NMR (400 MHz, CDCl3) 7.94–7.90 (d, 1H), 7.86-7.80 (d, 4H),7.52–7.42(m, 16H), 7.38(s, 2H), 7.18–7.12 (d, 2H), 5.87-5.94 (d, 3H), 5.52-5.47 (d, 2H). 13C NMR (400 MHz, CDCl3) δ=149.5, 148, 134, 130, 128, 127, 118, 98, 96, 54, 50, 49, 43, 26, 24; MALDI-TOF-MS(in DHB) of ADP(CA)_2_ : m/z calcd for C_54_H_32_BF_2_N_5_O_8_ : 927.67; found: 963.2[M+2(H_2_O)].

**ADP(CA)_2_**

**Scheme A** Synthetic methodology developed for ADP(CA)_2_.

**(2) Conjugation between Pluronic and PEG.** Using Pluronic-F98 and PEG-COOH (MW=30000) as an example, we describe the general procedure here. Solution I: We dissolved samples of 59.78 mg PEG-COOH and 10.36 mg Pluronic-F98 in 15 mL DMSO with gentle heating and vigorous shaking. Solution II: We dissolved DCC (14.93 mg) in 15 mL DMSO. Solution III: We dissolved DMAP (8.9 mg) in 15 mL DMSO. We transferred solution I into a three-neck flask and purged it with nitrogen for ten minutes. With nitrogen protection, we dropped solution II into the flask with a pressure-equalizing funnel (1 drop/second). In the same manner, we added solution III added to the mixture solution of I and II at a much slower dropping rate (1 drop/3 seconds). After that, the reaction took place in a nitrogen-protected environment for forty-eight hours at room temperature. We dialyzed the resulting solution with a dialysis tubing with 50K molecular-weight cutoff (Spectrum Laboratories Inc, Rancho Dominguez, CA) against DMSO and then water. We then collected, freeze-dried, and stored the purified sample at -20 °C for further use. The Pluronic F98 conjugating with PEG with MW of 20000, 30000 and 4000 represent as F98~PEG20K, F98~PEG30K and F98~PEG40K, respectively.

**(3) Synthesis of USF contrast agents.** a) ADP(CA)_2_-encapsulated Pluronic nanocapsules: we dissolved Pluronic F127 or F98 in deionized water (pH 8.5, w/v:5%) and dissolved the dye/TBAI (molar ratio = 1:6) in chloroform and kept it in sonication for thirty minutes. We then dropped the dye/TBAI chloroform solution into the Pluronic aqueous solution with agitation, further dispersed the solution with a sonicator (Qsonica, LLC., Newtown, CT, USA) at 20 W for four minutes, and kept the resulting solution stirring until the chloroform was completely evaporated. We collected the USF contrast agents by solution filtration using a 1.2 µm membrane (Fisher Scientific, Pittsburgh, PA, USA) and an Amicon Ultra centrifugal filter (10000 molecular weight cut-off, Millipore, Billerica, MA, USA). b) ICG-encapsulated PNIPAM NPs: the protocol was similar to our previous report [2]. In order to maintain the ICG-encapsulated nanoparticles’ stability, instead of ammonium persulfate (APS), we employed ACA as the initiator (0.070 g). We carried out the reaction at 70°C overnight in the absence of N,N,N’,N’-tetramethyl ethylene diamine (TEMED).

**References:**

1. Bandi V, El-Khouly ME, Ohkubo K, Nesterov VN, Zandler ME, Fukuzumi S, et al. Excitation-wavelength-dependent, ultrafast photoinduced electron transfer in bisferrocene/BF2-chelated-azadipyrromethene/fullerene tetrads. Chemistry. 2013;19(22):7221-30. doi: 10.1002/chem.201204317. PubMed PMID: 23554157.

2. Pei Y, Wei MY, Cheng B, Liu Y, Xie Z, Nguyen K, et al. High resolution imaging beyond the acoustic diffraction limit in deep tissue via ultrasound-switchable NIR fluorescence. Scientific reports. 2014;4:4690. doi: 10.1038/srep04690. PubMed PMID: 24732947; PubMed Central PMCID: PMC4003820.
