# Supplementary material for: High-Resolution Ultrasound-Switchable Fluorescence Imaging in Centimeter-Deep Tissue Phantoms with High Signal-To-Noise Ratio and High Sensitivity via Novel Contrast Agents
Source: PLoS One. 2016 Nov 9;11(11):e0165963. doi: 10.1371/journal.pone.0165963 (PMC5102469; doi:10.1371/journal.pone.0165963)
Supplement: S2 File — (DOCX) [file pone.0165963.s002.docx]

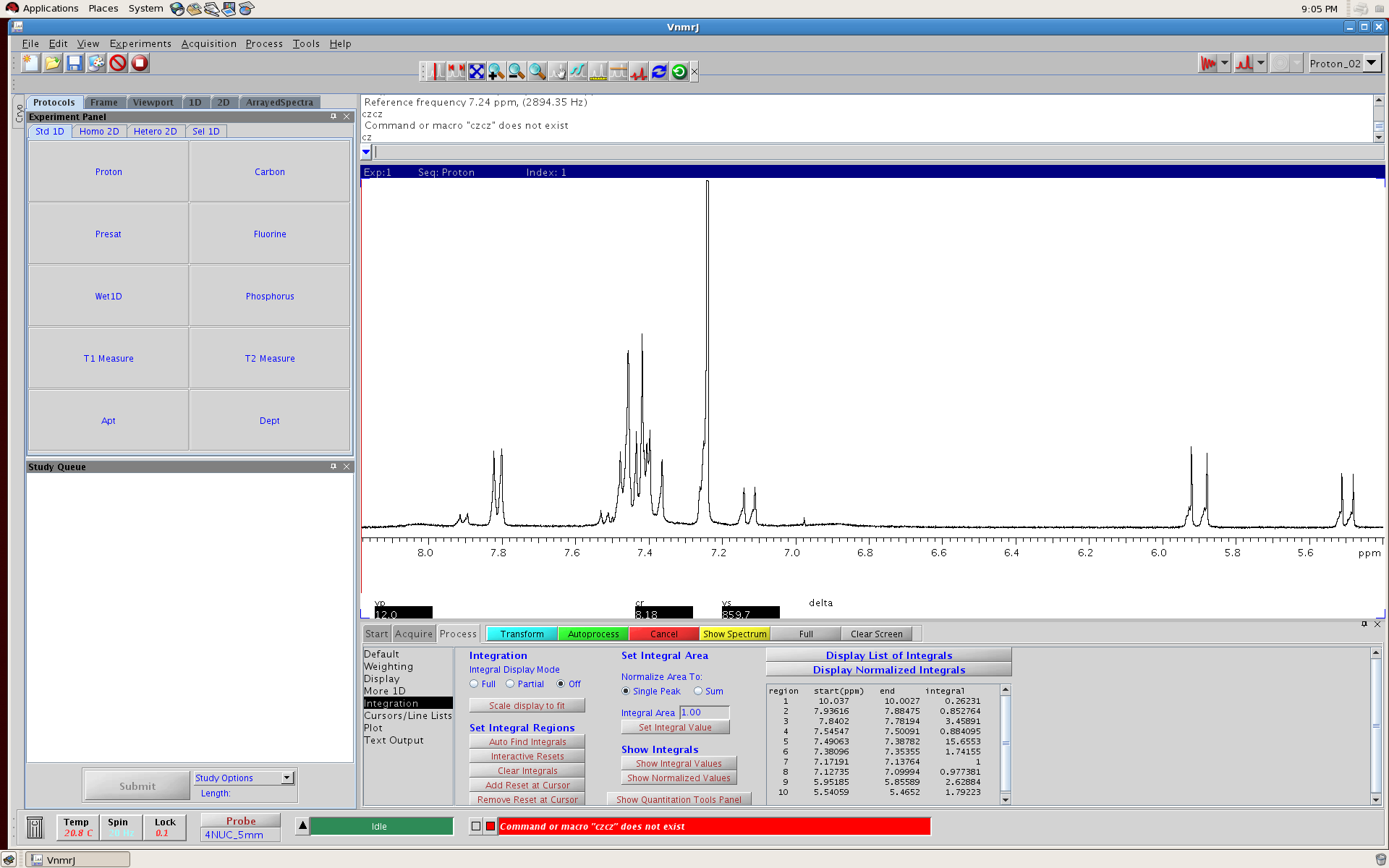


1H-NMR Spectra of compound ADP(CA)_2_ in CDCl_3_


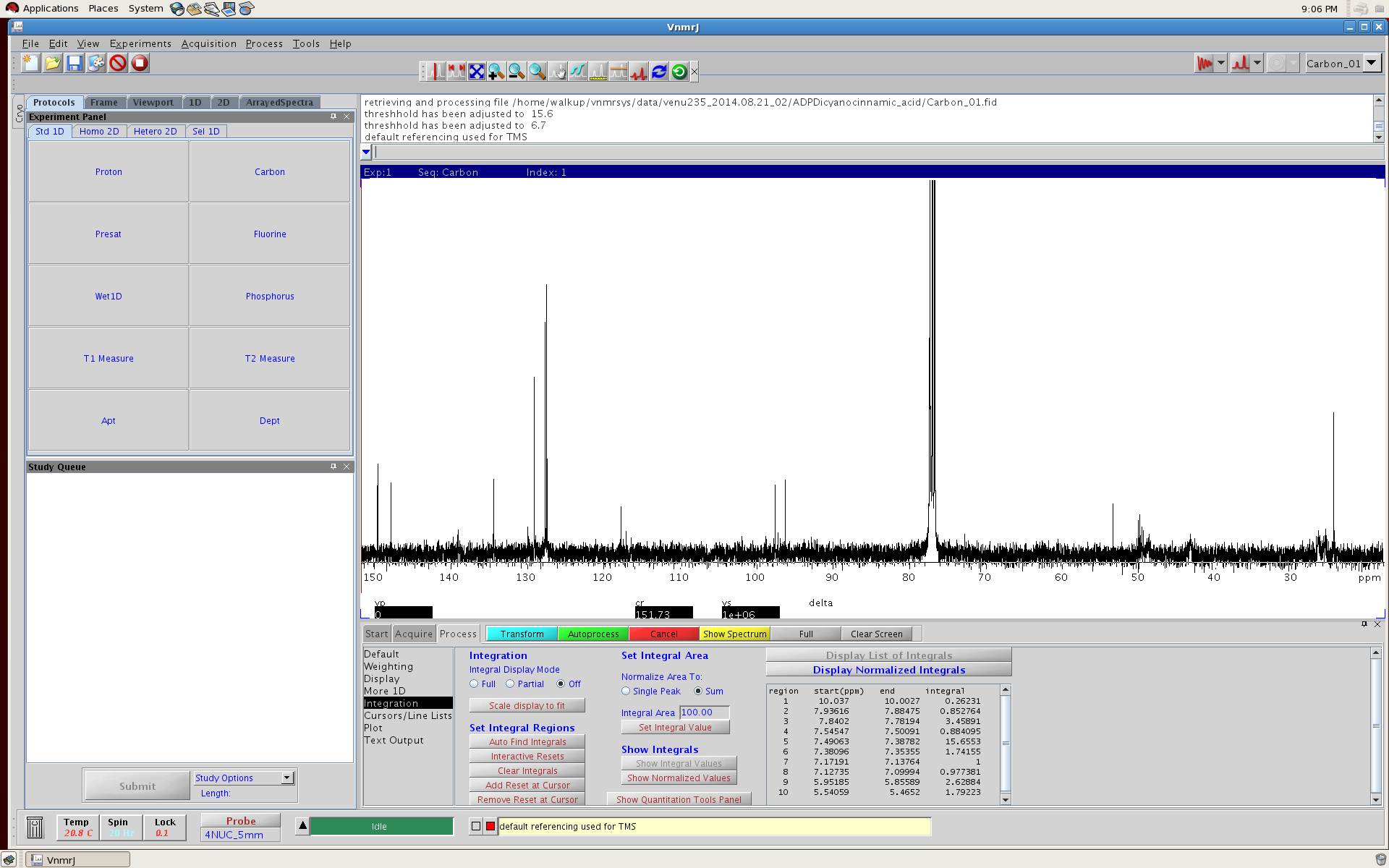


13C-NMR Spectra of compound ADP(CA)_2_ in CDCl_3_


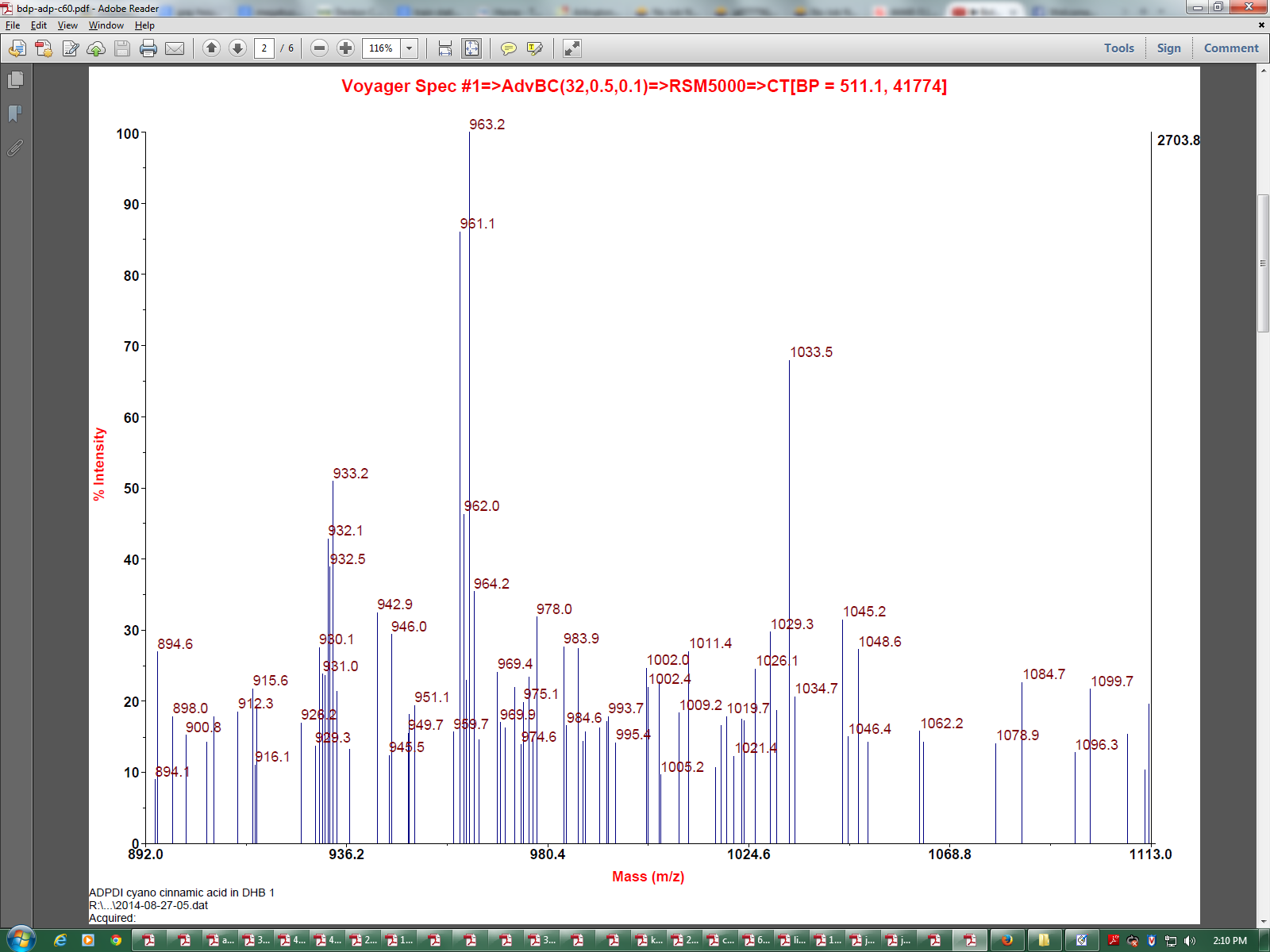


**Figure** **A** Nuclear magnetic resonance (NMR) and mass spectra of the ADP(CA)_2_.


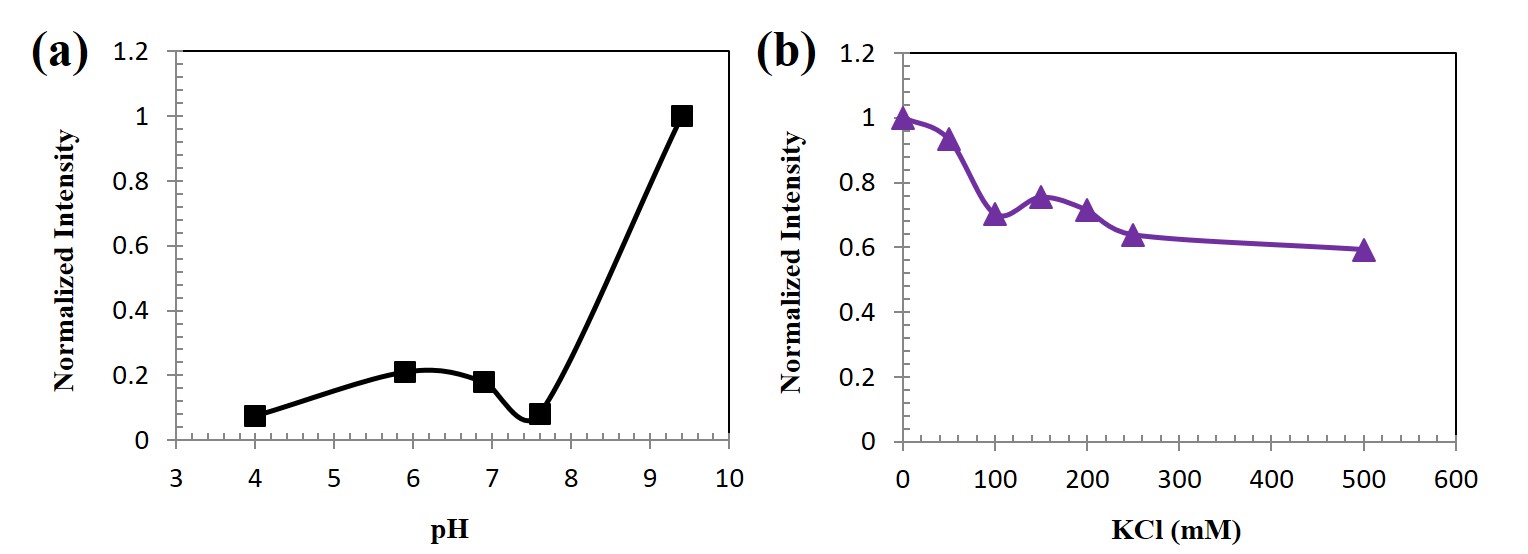


**Figure** **B** Fluorescence intensity of ADP(CA)_2_ aqueous solutions as a function of pH (a) and KCl concentration (b). Five solvents with different pH values were employed, which are: 4, 5.9, 6.9, 7.6 and 9.4. Seven solutions with different KCl concentration were tested, which are: 0, 50, 100, 150, 200, 250 and 500 mM. (a) Excitation: 655 nm; Filter: 711/25 band-pass filter; Laser power: 21.8 nJ. (b) Excitation filter: 632/22 band-pass filter; Emission filter: 711/25 band-pass filter.


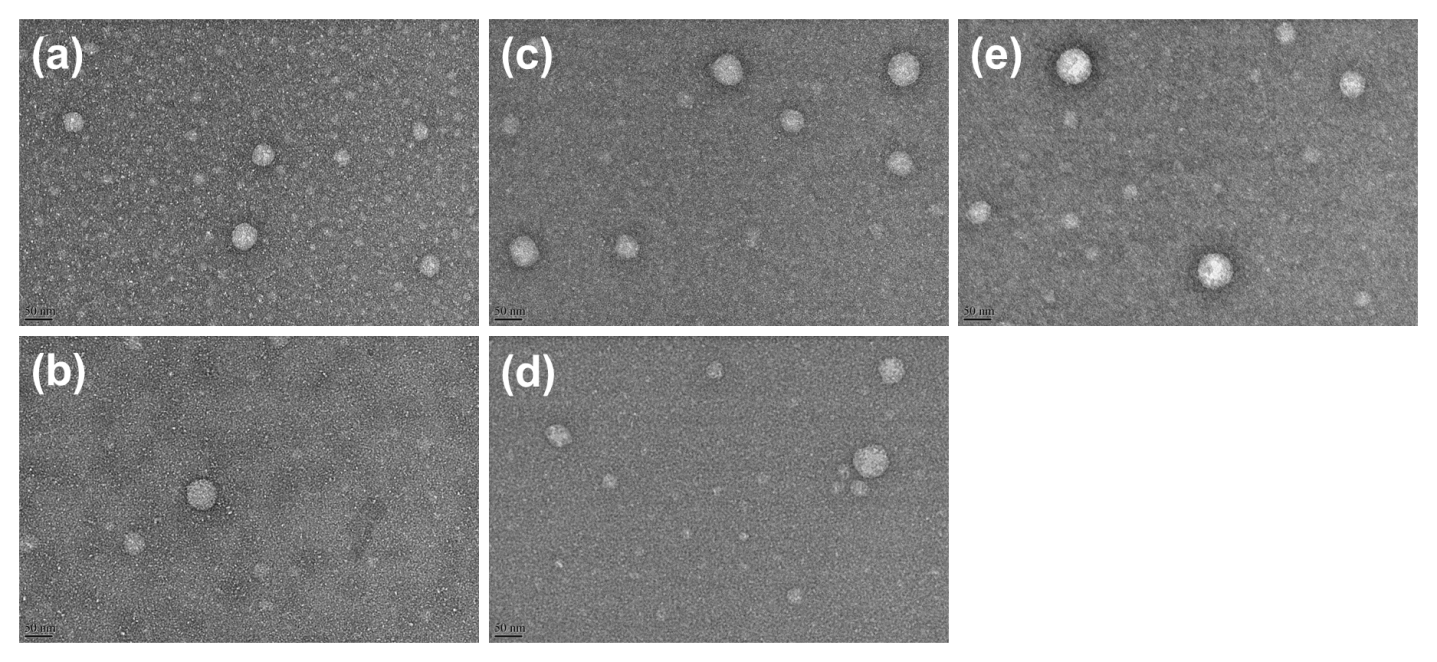


**Figure C** TEM images of dye-encapsulated nano-capsules: (a) F127, (b) F98, (c) F98~PEG20K, (d) F98~PEG30K, and (e) F98~PEG40K. Average diameter: (a) 26.0±6.4 (STD) nm, (b) 40.8±9.4 nm, (c) 48.7±10.9 nm, (d) 36.7±9.8 nm, and (e) 56.4±13.7 nm.

The size and morphology of the USF contrast agents were detected by transmission electron microscopy (TEM, JEOL 1200 EX, Peabody, MA, USA). Samples were prepared by casting an aqueous dispersion of nano-capsules (0.5~1 mg/mL) onto a carbon-coated copper grid (FF200-Cu-50, Electron Microscopy Sciences, Hatfield, PA, USA), followed by staining with 1% uranyl acetate (SPI-ChemTM, SPI, West Chester, PA, USA).

**
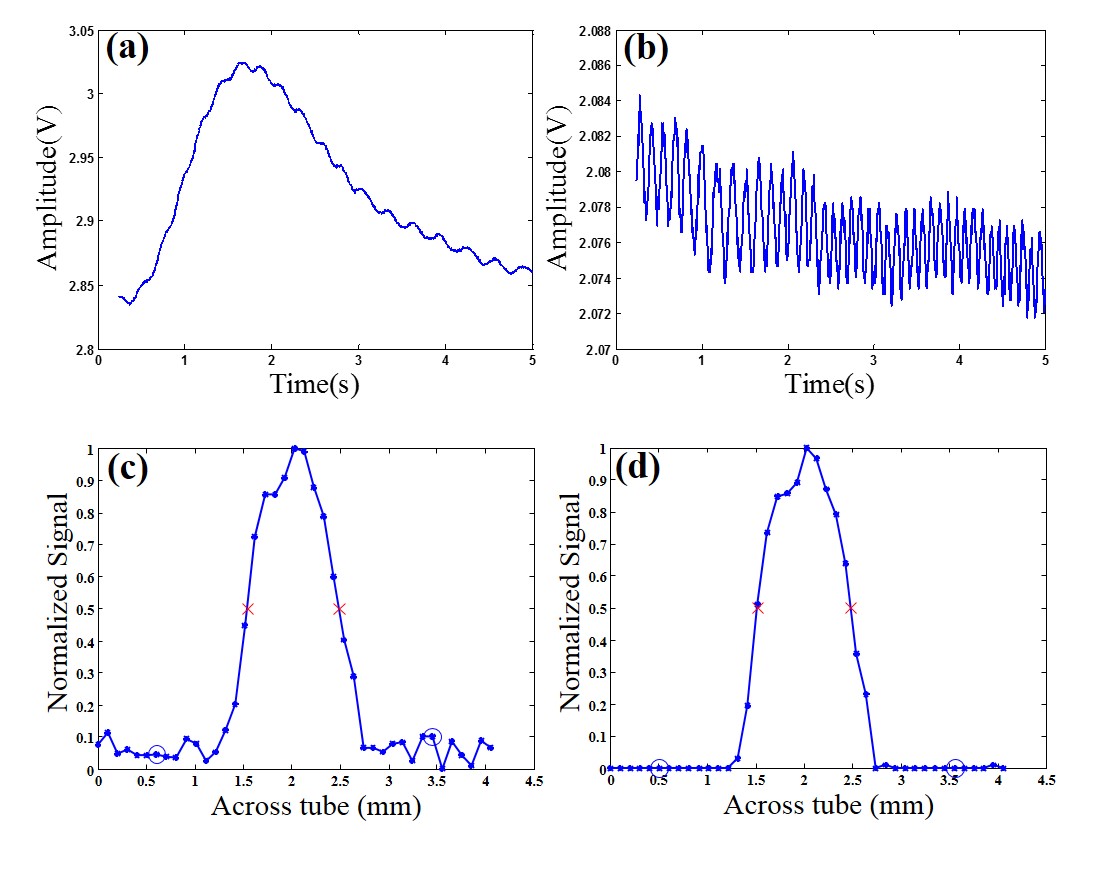
**

**Figure D** Signals related to ICG-encapsulated PNIPAM NPs in 8 mm-tissue, which shows (a) the typical USF signal, (b) the background noise signal, (c) the profile of one line in USF image before correlation (SNR: 31) and (d) the same profile after correlation (SNR: 345).
