# Supplementary material for: High-Resolution Ultrasound-Switchable Fluorescence Imaging in Centimeter-Deep Tissue Phantoms with High Signal-To-Noise Ratio and High Sensitivity via Novel Contrast Agents
Source: PLoS One. 2016 Nov 9;11(11):e0165963. doi: 10.1371/journal.pone.0165963 (PMC5102469; doi:10.1371/journal.pone.0165963)
Supplement: S3 File — (DOCX) [file pone.0165963.s003.docx]

**Effective signal identification algorithm**

Because background noise such as auto-fluorescence, laser leakage, or background fluorescence generated from fluorophores outside the ultrasound heating spot does not have the switching property, they therefore do not respond to the ultrasound pulse. Accordingly, all these noises do not have the USF signal’s unique temporal pattern (see Figs 3(a), (c–d)). Thus, the uniqueness of the USF signal’s shape provides an excellent indicator to differentiate them from other background noise. To differentiate USF signal from noise, we selected as reference one of the strongest USF signals at a specific location in an USF image (such as the signal in Fig 3(a) or Fig D(a) in S2 File). All the data acquired at other locations were correlated with this reference based on the equation (1). In the calibration experiments, known targets inside centimeter-deep tissues (including shape, position, and size) were imaged by FD-USF and processed by this algorithm.

After acquiring all USF data, we checked the raw data point by point and compared their curves with the calculated correlation coefficients to make sure they matched (e.g., all the signals in the background area show curves with no specific USF pattern and the calculated coefficients are less than 0.3; all the signals in the targeting area show curves with the USF pattern, and the corresponding coefficients are larger than 0.8). In addition, we knew the target’s shape, position, and true size. After intensive trials, we modified the algorithm until the processed image approximated the true case (shape, position, and size). After calibration, we finalized our algorithm and applied it to the experiments presented in this manuscript. We adopted the following strategies: (1) any signal with a CrC<0.3 was considered noise, and its USF strength (I_USF_) was set as zero; (2) any signal with a 0.3<CrC<0.8 was considered moderately possible to be a USF signal. Its USF strength was modified by multiplying the original I_USF_ with the cube of CrC (I_USF_×CrC^3^) to significantly suppress its level but still leave a certain contribution; (3) any signal with a CrC >0.8 was considered a USF signal and no modification was applied to its I_USF_. The results of the USF images this algorithm processed are accurate and can be verified by other imaging modalities such as ultrasound imaging and direct fluorescence imaging (see the section of “Multi-color high-resolution USF images in tissue-mimic silicone phantoms”).
