# Supplementary material for: High-Resolution Ultrasound-Switchable Fluorescence Imaging in Centimeter-Deep Tissue Phantoms with High Signal-To-Noise Ratio and High Sensitivity via Novel Contrast Agents
Source: PLoS One. 2016 Nov 9;11(11):e0165963. doi: 10.1371/journal.pone.0165963 (PMC5102469; doi:10.1371/journal.pone.0165963)
Supplement: S4 File — (DOCX) [file pone.0165963.s004.docx]

**Reason for choosing porcine muscle tissue**

Table A shows the optical properties of porcine muscle, human arm, and human breast tissue at excitation wavelength 671 nm. The optical properties of porcine muscle used in our experiments (u_a_ and u_s_’) are nearly twice less than those of a human arm. However, because our current laser intensity (0.21 mW/cm^2^ when imaging depth <3 cm) is well below the ANSI safety threshold, we can increase the laser energy to compensate for the signal attenuation caused by large tissue absorption and scattering coefficients that occurs when we perform USF on living animals or human beings. To characterize the required laser enhancement, we performed a Monte Carlo simulation, the results of which Fig A displays. For instance, to gain the same fluence at different depth in arm tissue, one can increase the laser energy 1.61-fold at 1 cm, 1.32-fold at 2 cm, and 1.35-fold at 3 cm. Similarly, when imaging breast tissues, one should increase the laser energy 1.26-fold at 1 cm, 1.35-fold at 2 cm, and 1.33-fold at 3 cm.

Note that another reason that we selected porcine muscle tissue as the tissue phantom for USF imaging is due to its acoustic property. In ultrasound-related studies such as photoacoustic imaging [1], sonography [2], and intense ultrasound energy delivery [3], porcine muscle tissue was frequently used.

**Table A** Optical property summary

| **Sample** | **µ_a_ (cm^-1^)** | **µ_s_^'^ (cm^-1^)** |
| --- | --- | --- |
| Porcine muscle | 0.06 | 2.7 |
| Human arm | 0.12 | 5.8 |
| Human breast ^[4]^ | 0.037-0.11 | 11.4-13.5 |


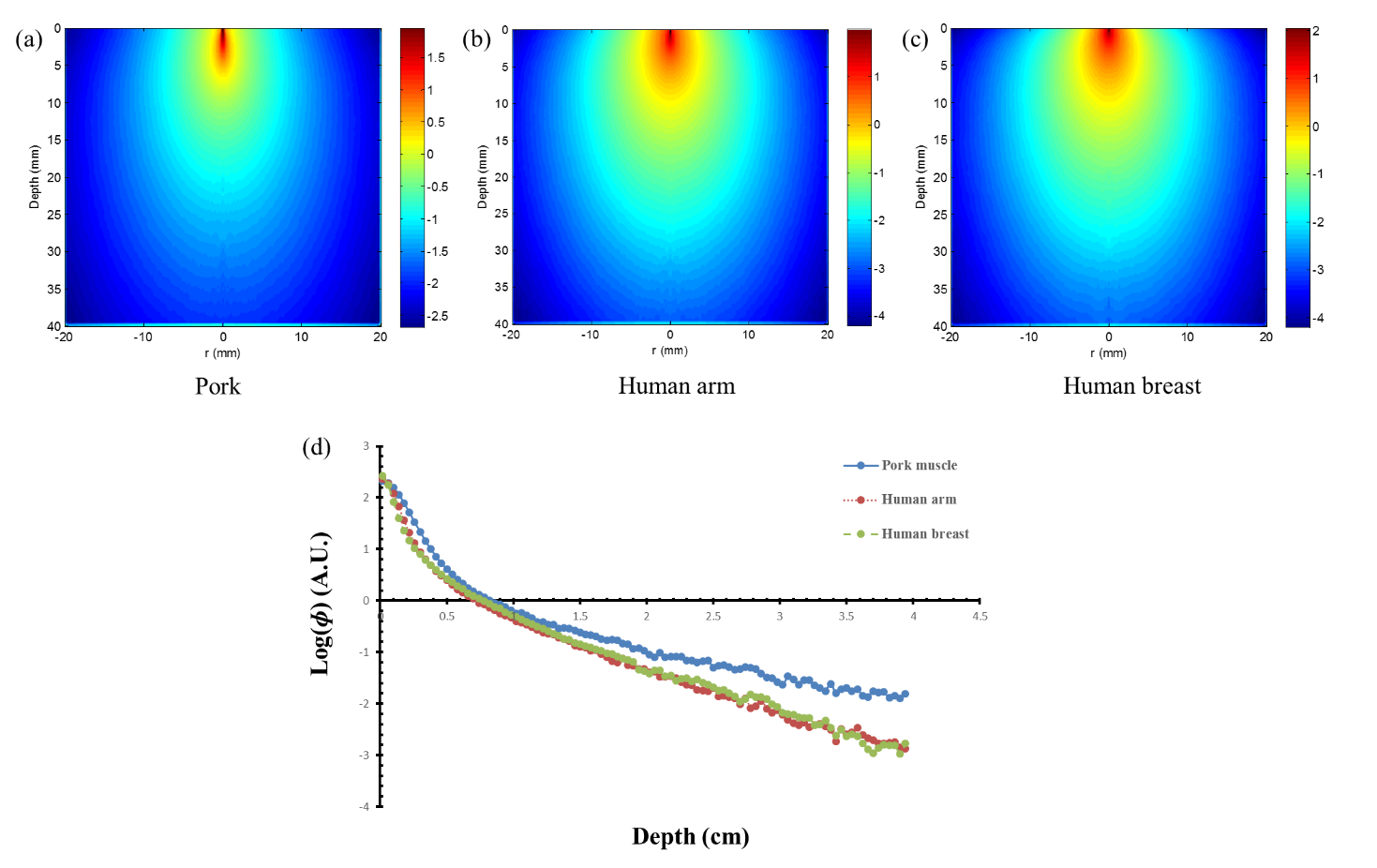


**Figure A** Monte Carlo simulation of (a) pork muscle (b) human arm and (c) human breast. The parameters used in this simulation are: µ_a_: 0.06, 0.12 and 0.06 cm^-1^, µ_s_': 2.7, 2.8 and 12 cm^-1^ and g: 0.9, 0.9 and 0.9 in an order of (a), (b) and (c). (d) plotted the relationship between fluence and penetration depth.

**References:**

1. Shah J, Park S, Aglyamov S, Larson T, Ma L, Sokolov K, et al. Photoacoustic imaging and temperature measurement for photothermal cancer therapy. J Biomed Opt. 2008;13(3):034024. doi: 10.1117/1.2940362. PubMed PMID: 18601569; PubMed Central PMCID: PMCPMC2713867.

2. Dupont AC, Sauerbrei EE, Fenton PV, Shragge PC, Loeb GE, Richmond FJ. Real-time sonography to estimate muscle thickness: comparison with MRI and CT. J Clin Ultrasound. 2001;29(4):230-6. doi: 10.1002/jcu.1025. PubMed PMID: 11323778.

3. White WM, Makin IRS, Slayton MH, Barthe PG, Gliklich R. Selective transcutaneous delivery of energy to porcine soft tissues using Intense Ultrasound (IUS). Laser Surg Med. 2008;40(2):67-75. doi: 10.1002/lsm.20613. PubMed PMID: WOS:000253670900002.

4. Sandell JL, Zhu TC. A review of in-vivo optical properties of human tissues and its impact on PDT. J Biophotonics. 2011;4(11-12):773-87. doi: 10.1002/jbio.201100062. PubMed PMID: WOS:000297740500001.
