# Supplementary material for: High-Resolution Ultrasound-Switchable Fluorescence Imaging in Centimeter-Deep Tissue Phantoms with High Signal-To-Noise Ratio and High Sensitivity via Novel Contrast Agents
Source: PLoS One. 2016 Nov 9;11(11):e0165963. doi: 10.1371/journal.pone.0165963 (PMC5102469; doi:10.1371/journal.pone.0165963)
Supplement: S5 File — (DOCX) [file pone.0165963.s005.docx]

**Further discussion about future directions**

The synthesis yields of the high-threshold (T_th1_>37 ^°^C) USF contrast agents are low (<7%) because of the low conjugation efficiency of Pluronic-F98 with PEG-COOH and the high cost of commercially available PEG-COOH. In addition, reducing the synthesis time and stabilizing the agents are also necessary. Also necessary is optimizing current synthesis strategies or developing new synthesis protocols for the high-threshold (T_th1_>37^°^ C) USF contrast agents, such as converting the OH group in the Pluronic polymer into COOH group and then conjugating with PEG-NH_2_ to increase the conjugation efficiency (currently it is in progress in our lab).

Exploring more USF fluorophores to cover the NIR spectrum of 750–900 nm will be useful for achieving even deeper tissue imaging, because tissue has relatively lower absorption and scattering coefficients in this range (compared with 670–700 nm). Also, the ability to synthesize more NIR USF fluorophobes is useful for *in vivo* and multi-color USF imaging. Currently, we are investigating other dyes in the same family in our laboratories.

The axial size of a single-element HIFU transducer is always a few times longer than the lateral size, which will degrade the quality of 3-D images. Adopting two 90°-crossed transducers with overlapped foci may partially solve this issue. We are currently investigating this strategy in our laboratories (results to be published elsewhere). In addition, adopting a transducer with a small f-number <1 is also helpful, because the axial focal size is proportional to the square of the f-number.

Improving USF photon collection efficiency can further increase the current system’s sensitivity and further increase the SNR and imaging depth or reduce the adopted concentration. One possible way is to adopt a cooled and intensified CCD camera—or even multiple cameras—to collect more USF photons from all directions in the tissue because USF photons are highly scattered and propagate outside tissue in all directions. Increasing the detection area can increase collection of more USF photons and improve sensitivity. A time-gating method may effectively block background laser leakage and non-USF fluorescence photons, thus further increasing system sensitivity, and is currently under investigation in our laboratories (results to be published elsewhere).

Last, the current imaging system is not suitable for scanning live animals because of the irregular proportions of animals’ bodies and motions and the limited imaging speed. With significantly improved imaging speed (as discussed above), designing a system with the following features will be very useful for *in vivo* studies and highly possible: (1) a large photon collection area (such as a cooled and intensified CCD camera); (2) electronically scanning ultrasound focus without moving the animal; and (3) possibly synchronizing the imaging system with the animal’s physiological motions.
