# Supplementary material for: High-Resolution Ultrasound-Switchable Fluorescence Imaging in Centimeter-Deep Tissue Phantoms with High Signal-To-Noise Ratio and High Sensitivity via Novel Contrast Agents
Source: PLoS One. 2016 Nov 9;11(11):e0165963. doi: 10.1371/journal.pone.0165963 (PMC5102469; doi:10.1371/journal.pone.0165963)
Supplement: S1 Table — λex is the wavelength of the excitation laser in the measurements (655 nm), and λem is the central wavelength of the band pass emission filter. The number of 25 in the parentheses is the bandwidth of the emission filter. The definitions of the other three parameters are the same as those in our previous publication3. (DOCX) [file pone.0165963.s006.docx]

**Table S1** Summary of USF contrast agents. λ_ex_is the wavelength of the excitation laser in the measurements (655 nm), and λ_em_ is the central wavelength of the band pass emission filter. The number of 25 in the parentheses is the bandwidth of the emission filter. The definitions of the other three parameters are the same as those in our previous publication [1] .

| **Pluronic based**  **Polymers** | **λ_ex_, λ_em_**  **_(nm)_** | **I_On_/I_off_** | **T_th1_**  **_(˚C)_** | **T_BW_**  **_(˚C)_** |
| --- | --- | --- | --- | --- |
| F127 | 655, 711(25) | 362 | 23 | 4.5 |
| F98 | 655, 711(25) | 352.4 | 30.5 | 6.5 |
| F98~PEG20K | 655, 711(25) | 272.1 | 35.5 | 14.7 |
| F98~PEG30K | 655, 711(25) | 211.6 | 45.6 | 10 |
| F98~PEG40K | 655,711 (25) | 287.6 | 45.2 | 6.8 |

**References:**

1. Cheng BB, Wei MY, Liu Y, Pitta H, Xie ZW, Hong Y, et al. Development of Ultrasound-Switchable Fluorescence Imaging Contrast Agents Based on Thermosensitive Polymers and Nanoparticles. Ieee J Sel Top Quant. 2014;20(3). doi: Artn 6801214, Doi 10.1109/Jstqe.2013.2280997. PubMed PMID: ISI:000329998200006.
